# Effect of Differential Speed Ratio on the Microstructural Evolution and Mechanical Properties of Asynchronously Rolled 7075 Aluminum Alloy

**DOI:** 10.3390/ma19071412

**Published:** 2026-04-01

**Authors:** Lanshun Wei, Xiaowei Lian, Liping Deng, Bingshu Wang

**Affiliations:** 1 School of Advanced Manufacturing, Fuzhou University, Fuzhou 362200, China; 2 College of Materials Science and Engineering, Fuzhou University, Fuzhou 350108, China

**Keywords:** 7075 aluminum alloy, differential speed rolling, microstructure, mechanical properties

## Abstract

The increasing demands of application conditions urgently call for process innovations in high-performance 7xxx aluminum alloys. This study investigated the effect of differential speed rolling (DSR) on the microstructural evolution and mechanical properties of 7075 aluminum alloy subjected to DSR with a total reduction of 60%, followed by isothermal aging at 120 °C for 24 h. The results show that DSR promotes the development of grain refinement, defect accumulation, and deformation texture, while the corresponding strengthening effect exhibits a non-monotonic dependence on speed ratio. Among all conditions, the DSR2.0 sample exhibits the most favorable microstructure, characterized by the highest kernel average misorientation (KAM) value, the strongest deformation texture, and the finest as well as most densely distributed intragranular η′ precipitates. Accordingly, the DSR2.0 sample achieves the optimal strength–ductility balance, with a yield strength, ultimate tensile strength, elongation, and hardness of 582.26 MPa, 648.43 MPa, 10.75%, and 199.8 HV, respectively. Specifically, the deterioration in the properties of the DSR2.5 sample is attributed to localized recovery, shear inhomogeneity and coarsening of precipitates. The differential speed ratio enables effective optimization of the 7075 aluminum alloy by regulating the evolution of grains, dislocations, precipitate phases, and texture, among which precipitation strengthening is the dominant calculated contribution. Therefore, an appropriate differential speed ratio is key to achieving performance optimization.

## 1. Introduction

Al-Zn-Mg-Cu alloys are an important class of lightweight structural materials and have been widely used in the automotive, construction, and aerospace fields due to their high specific strength, good workability, and excellent corrosion resistance [1,2,3]. With the rapid development of manufacturing industries and the increasing demands of service conditions, higher requirements have been imposed on the synergistic optimization of strength, ductility, and microstructural stability in aluminum alloys. Over the past few decades, severe plastic deformation (SPD) techniques, such as equal-channel angular pressing (ECAP), high-pressure torsion (HPT), differential speed rolling (DSR), and accumulative roll bonding (ARB), have been extensively investigated for grain refinement and strength enhancement in aluminum alloys [4,5,6].

Among the various SPD routes, differential speed rolling (DSR) is a typical asymmetric rolling process, in which additional shear deformation is introduced by the velocity difference between the upper and lower rolls, thereby generating a more uniform plastic strain distribution through the sheet thickness. Previous studies have demonstrated that DSR is effective in promoting grain refinement, regulating texture evolution, and improving microstructural homogeneity. Compared with symmetric rolling, DSR can reduce the rolling force to a certain extent and effectively tailor the microstructure and mechanical properties of sheets through the additional shear deformation imposed across the thickness direction [7]. Moreover, rolling under a high-speed ratio generally results in a finer and more homogeneous microstructure, together with more pronounced shear texture components [8].

To date, a series of studies have been conducted on the effect of DSR on the microstructural evolution and property response of aluminum alloys. Compared with symmetric rolling, asynchronously rolled 6061 aluminum alloy exhibits a lamellar subgrain structure surrounded by low-angle grain boundaries. Magalhães et al. [9] reported that artificial aging after symmetric rolling at room temperature was more effective in improving strength and hardness, whereas cryogenic asynchronous rolling followed by the same post-aging treatment increased uniform elongation and reduced texture intensity. Amir et al. [10] found that the grain width of 7075 aluminum alloy decreased significantly after asynchronous rolling with a thickness reduction of 60%, and distinct shear bands were observed on the RD-ND plane at reductions of 40% and 60%. Wang et al. [11] further reported that differential speed ratio rolling markedly altered the grain structure, texture characteristics, and mechanical response of Al-Cu-Li-TiC/TiB_2_ alloys, highlighting the significant influence of shear deformation on microstructural evolution and through-thickness heterogeneity. More recent studies have shown that the role of differential speed ratio rolling is not limited to a simple increase in strength. Byrska-Wójcik et al. [12] demonstrated that differential speed ratio rolling with tilted material entry could effectively regulate the through-thickness texture distribution of aluminum sheets and improve the uniformity of mechanical response. In addition, Ehsani et al. [13] showed that differential speed ratio reverse rolling of AA5052 alloy promoted deformation band formation, grain subdivision, and continuous dynamic recrystallization, thereby achieving superior strength–ductility synergy. However, the effect of speed ratio cannot be regarded as an isolated variable. Shao et al. [14] pointed out that rolling temperature and speed ratio may jointly affect deformation coordination, interfacial evolution, and the final microstructure during differential speed ratio rolling. Therefore, for specific alloy systems, the intrinsic mechanism of DSR still requires further clarification.

In summary, although previous studies have demonstrated that symmetric/differential speed ratio rolling can significantly modify the microstructure, texture characteristics, and mechanical properties of aluminum alloys, a systematic understanding of the coupling among grain refinement, dislocation accumulation, texture evolution, precipitation behavior, and mechanical response under different speed-ratio conditions is still lacking for precipitation-hardenable 7075 aluminum alloy. Therefore, the present work systematically investigates the effect of differential speed ratio on the microstructural evolution and mechanical properties of asynchronously rolled 7075 aluminum alloy, with emphasis on grain structure, dislocation characteristics, texture development, and intragranular precipitation behavior. By combining these results with strengthening analysis, this study clarifies the role of the differential speed ratio in controlling the comprehensive properties of 7075 alloy. The main contributions of this work are as follows: the non-monotonic evolution of microstructure and properties with an increasing speed ratio is identified; DSR2.0 is determined to be the critical condition for optimized comprehensive performance; and the intrinsic relationships among speed ratio, precipitation characteristics, texture intensity, and strengthening contributions are established.

## 2. Materials and Methods

### 2.1. Materials and Processing

A rolled thick plate of 7075 aluminum alloy was used as the starting material in this study. The initial dimensions of the plate were 400 mm rolling direction (RD) × 300 mm transverse direction (TD) × 20 mm normal direction (ND). The chemical composition of the alloy is listed in Table 1. The Inductively Coupled Plasma Optical Emission Spectrometry (ICP-OES) results indicate that the alloy composition meets the relevant standard requirements for 7075 aluminum alloy.

To clarify the independent effect of differential speed ratio on the microstructural evolution and mechanical properties of 7075 aluminum alloy, symmetric rolling (SR) and DSR were performed under identical solution treatment and quenching conditions to prepare comparative samples with different speed ratios. The alloy was solution-treated at 470 °C for 90 min, followed by water quenching to obtain the solution-treated (ST) sample. During quenching, the transfer time was strictly controlled within 5 s. To minimize the influence of natural aging on the subsequent comparison of microstructure and properties, the interval between quenching and rolling was strictly controlled within 2 h. After rolling, all samples were subjected to the same single-stage isothermal aging treatment at 120 °C for 24 h. It should be noted that, because the dislocation density, stored energy, and defect structure vary with differential speed ratio, the precipitation kinetics and the time required to reach the peak-aged state are not identical for all samples. Therefore, the present results should be interpreted as a comparison of different DSR conditions under the same aging schedule, rather than a comparison of individually optimized peak-aged samples.

To ensure a comparable cumulative reduction among samples processed at different speed ratios, the initial specimen thickness was fixed at 4 mm, and the total reduction was set at 60%. A multi-pass rolling route was adopted to achieve the target reduction, with an individual pass reduction of approximately 10%. The differential speed ratio (*S*) was defined as the ratio of the linear velocities of the upper (*v_upper_*) and lower (*v_lower_*) work rolls, as shown in Equation (1):

(1) $$\begin{array}{c} S=v_{upper}/v_{lower} \end{array}$$

**Figure 1:**
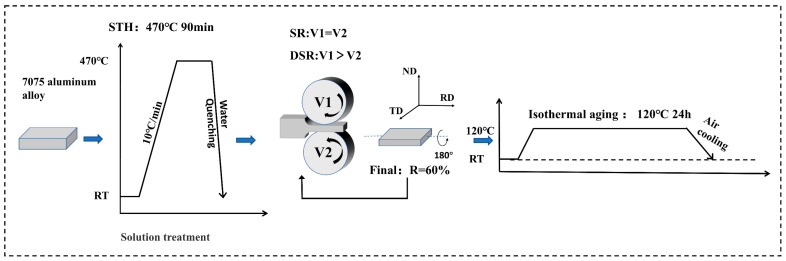
The process roadmap used in this study.

Rolling was carried out on a four-high reversing rolling mill (YS-FW360AR, Qixinshidai Technology Co., Ltd., Tianjin, China) equipped with work rolls of Φ120 mm. In the SR condition, both the upper and lower work rolls were operated at 10 r/min. In the DSR condition, different speed ratios were achieved by increasing the rotational speed of the upper work roll, while the lower roll speed was kept constant. The speed ratios used in this study were 1.5, 2.0, and 2.5, corresponding to upper-roll speeds of 15, 20, and 25 r/min, respectively. During rolling, the reduction in each pass, roll speed, final thickness, and sheet shape were recorded, and the rolled samples were labeled according to the designation rule “SR/DSR1.5”. All rolling experiments were conducted at room temperature. The sample temperature was measured within 5 s after each rolling pass using an infrared thermometer, with no fewer than three positions measured for each sample. The average temperatures measured after a single rolling pass were 36.7 ± 4.3 °C, 52.4 ± 5.7 °C, 71.8 ± 6.4 °C, and 88.6 ± 5.9 °C for the SR, DSR1.5, DSR2.0, and DSR2.5 samples, respectively. After each pass, the sheets were allowed to cool naturally in the air to room temperature before the next pass. During rolling, a small amount of lubricating oil was uniformly applied to the roll surface only before the first pass of each sample. The same conventional lubrication condition was maintained for all samples to minimize the influence of frictional variations on the additional shear deformation and to avoid material transfer or sticking on the roll surface. The overall processing route is illustrated in Figure 1. The aging treatment was carried out in a resistance furnace (KSL-1200X-J, Hefei Kejing Materials Technology Co., Ltd., Hefei, China), with the temperature deviation controlled within ±2 °C.

### 2.2. Microstructure Characterization and Properties Tests

The microstructure of the 7075 aluminum alloy was characterized using a field-emission scanning electron microscope (FE-SEM, Regulus 8100, Hitachi, Tokyo, Japan) and a field-emission scanning electron microscope equipped (Zeiss Supra 55, Carl Zeiss AG, Oberkochen, Germany) with an electron backscatter diffraction (EBSD) system (NordlysMax2, Oxford Instruments, Oxford, UK). The EBSD specimens were prepared from the rolled and subsequently aged samples, and the scanning step size was set to 0.5 μm. Data analysis was performed using AZtecCrystal software (version 2.1) (For the ST sample, the EBSD scan area was 450 μm × 450 μm, and approximately 500 grains were indexed. For the rolled samples, the EBSD scan area was 430 μm × 350 μm, and approximately 800 grains were analyzed). An X-ray diffractometer (XRD, Rigaku Miniflex 600, Rigaku Corporation, Tokyo, Japan) with Cu Kα radiation was employed to identify the crystal structure and phase constituents in the 2θ range of 20–90°, and the dislocation density was quantitatively estimated based on diffraction peak broadening. The tensile fracture surfaces were further examined by scanning electron microscopy (SEM, Regulus 8100, Hitachi, Tokyo, Japan). Transmission electron microscopy (TEM) was used for further characterization of the intragranular and grain-boundary microstructures. Bright-field TEM images and selected-area electron diffraction (SAED) patterns were acquired using a transmission electron microscopy (FEI Tecnai F20, Thermo Fisher Scientific, Waltham, MA, USA) operated at 200 kV.

According to the ASTM E8/E8M-04 (standard) [15], dog-bone-shaped tensile specimens were machined by wire electrical discharge cutting from the rolled and aged samples along the RD, with a total length of 25 mm and a gauge length of 6 mm. Room-temperature tensile tests were carried out on a universal testing machine (MTS Exceed E45, MTS Industrial Systems (China) Co., Ltd., Shanghai, China) at a crosshead speed of 0.2 mm/min. For each processing condition, five parallel specimens were tested to obtain the average stress–strain curves, ultimate tensile strength (UTS), yield strength (YS), and elongation to fracture (EL). Vickers microhardness measurements were performed on the RD-TD plane under a load of 200 gf and a dwell time of 15 s. At least eight indentations were measured for each sample.

## 3. Results and Discussion

### 3.1. Microstructure and Texture Characteristics of the Solution-Treated Condition

**Figure 2:**
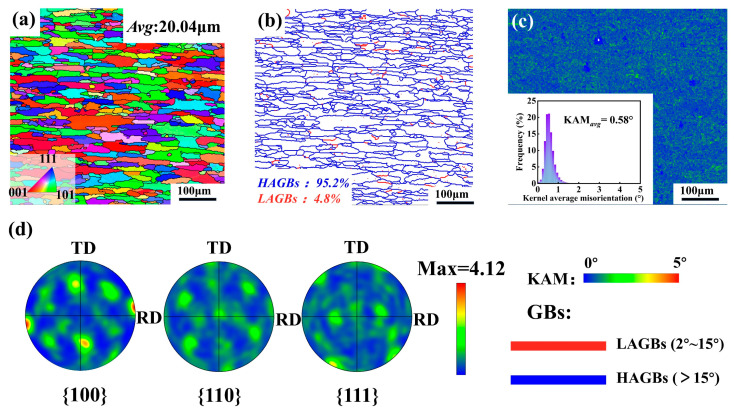
Microstructural characteristics of the 7075 aluminum alloy in the ST condition: (**a**) IPF map; (**b**) grain boundary distribution; (**c**) KAM map; and (**d**) pole figures.

Figure 2 presents the microstructural characteristics of the ST sample. The IPF map shows that the grains in the ST condition are elongated along the RD and gradually tend toward an equiaxed morphology, with an average grain size of ~20.04 μm (Figure 2a). In addition, the high fraction of high-angle grain boundaries (HAGBs, 95.2%) and the low average KAM value (0.58°) indicate a relatively high degree of recrystallization (Figure 2b,c). This provides a relatively stable initial microstructural state for the subsequent deformation and aging treatments. As shown in Figure 2d, the pole figures reveal a pronounced <100>//RD orientation in the ST sample, further confirming the relatively sufficient recrystallization.

### 3.2. Microstructure and Texture Evolution of 7075 Aluminum Alloy at Differential Speed Ratios

**Figure 3:**
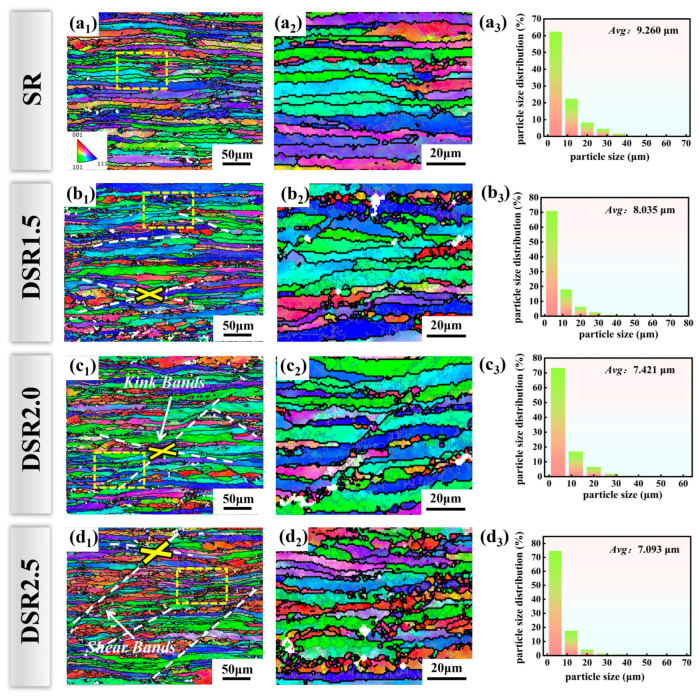
IPF maps and grain size statistics of 7075 aluminum alloy at differential speed ratios: (**a_1_**–**d_1_**) overall IPF maps; (**a_2_**–**d_2_**) enlarged local IPF maps; and (**a_3_**–**d_3_**) grain size distributions.

Figure 3 presents the overall EBSD inverse pole figure (IPF) maps, enlarged local views, and grain size statistics of 7075 aluminum alloy processed at differential speed ratios under the same total reduction. As shown in Figure 3(a_1_–d_1_), all rolled samples exhibit grains markedly elongated along the RD, forming a typical banded deformation structure. Compared with the SR sample, the deformation bands in the DSR samples become increasingly tortuous and undulated. The enlarged views in Figure 3(a_2_–d_2_) further show that the grain boundaries in the SR sample remain relatively straight and the banded structure is more continuous. In contrast, with increasing differential speed ratio, the grain boundaries become progressively fragmented and segmented, and fine grains or subgrains locally appear within the deformation bands.

Under the DSR1.5 and DSR2.0 conditions, typical kink bands (KBs) and shear bands (SBs) can be clearly identified, indicating that shear localization becomes more pronounced at intermediate differential speed ratios [16]. This behavior can be attributed to the enhanced frictional asymmetry and shear strain imposed by the higher roll speed mismatch during DSR [17]. In the DSR2.5 sample, the traces of shear bands become more distinct, and the banded structure is further disrupted by intense shear deformation.

The grain size statistics are shown in Figure 3(a_3_–d_3_). With an increasing differential speed ratio, the average grain size decreases progressively from 9.260 μm in the SR sample to 7.093 μm in the DSR2.5 sample, accompanied by a gradual shift in the grain size distribution toward the finer-size range. This result indicates that the additional shear strain introduced by differential speed rolling promotes dislocation multiplication and substructure refinement, thereby accelerating grain subdivision. However, the grain refinement effect becomes less pronounced at higher speed ratios, suggesting that the refinement tendency gradually approaches saturation.

**Figure 4:**
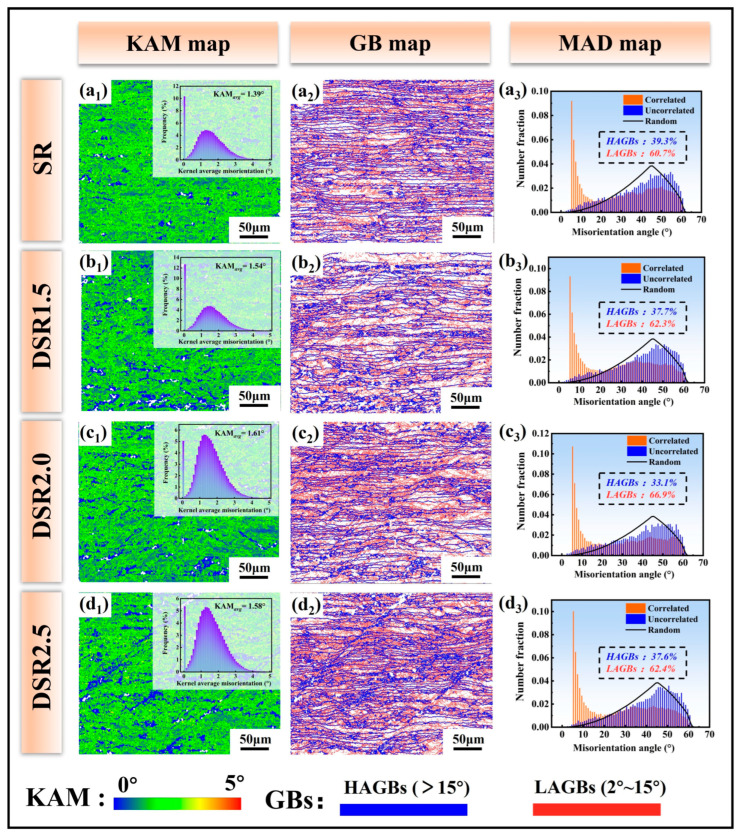
KAM maps, grain boundary distribution maps, and misorientation angle distributions of 7075 aluminum alloy at differential speed ratios: (**a_1_**–**d_1_**) KAM maps; (**a_2_**–**d_2_**) grain boundary distribution maps; and (**a_3_**–**d_3_**) misorientation angle distribution maps.

Figure 4 shows the kernel average misorientation (KAM) distributions, grain boundary characteristic maps, and misorientation angle distribution (MAD) profiles of 7075 aluminum alloy processed at differential speed ratios under the same total reduction. As shown in Figure 4(a_1_–d_1_), the KAM distributions of all samples exhibit a banded morphology elongated along the RD. Since the KAM value can, to some extent, reflect the intragranular dislocation density [18], it provides useful information on the evolution of local strain accumulation and stored energy during rolling. With increasing differential speed ratio, the average KAM value generally increases and reaches a maximum of 1.61° in DSR2.0, indicating that redundant shear deformation effectively promotes defect accumulation at an appropriate speed ratio. When the differential speed ratio is further increased to 2.5, the KAM value decreases slightly, suggesting that the incremental effect of defect storage becomes less pronounced. Considering the limited spatial resolution and sampling-area sensitivity of EBSD [19], XRD analysis was further employed in Section 3.5 to quantitatively evaluate the overall dislocation density.

Rolling deformation generally introduces a high density of dislocations, and their re-arrangement and entanglement during recovery promote the formation of subgrains and low-angle grain boundaries (LAGBs) [20]. As shown in Figure 4(a_2_–d_2_), all processed samples are dominated by LAGBs. With increasing differential speed ratio, the fraction of LAGBs first increases and reaches its highest level in DSR2.0, while the HAGB fraction shows an opposite trend.

When the differential speed ratio is further increased to 2.5, the fraction of LAGBs decreases slightly, accompanied by a corresponding increase in the HAGB fraction. Together with the slight reduction in KAM value, this indicates that excessive speed ratio weakens defect accumulation and is accompanied by partial dislocation rearrangement or local recovery. The MAD curves of all samples show pronounced peaks in the low-angle range, further confirming that the microstructural evolution under the present rolling conditions is mainly dominated by dislocation organization and subgrain formation.

**Figure 5:**
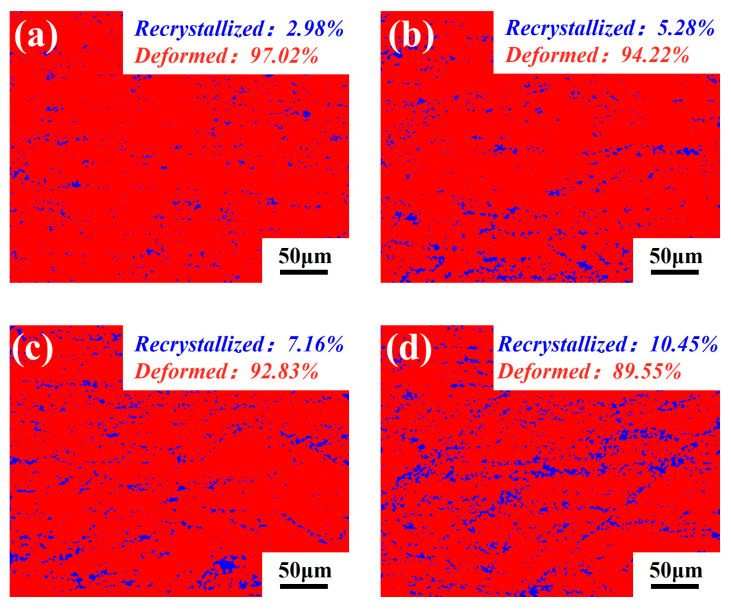
Recrystallized microstructure distributions of 7075 aluminum alloy processed at differential speed ratios: (**a**) SR; (**b**) DSR1.5; (**c**) DSR2.0; and (**d**) DSR2.5.

Figure 5 shows the distribution of recrystallized microstructures in 7075 aluminum alloy processed at differential speed ratios. Overall, all samples are still dominated by deformed structures, with a relatively low recrystallized fraction and substantial retained deformation substructures. With increasing differential speed ratio, the recrystallized fraction generally increases and reaches a maximum of 10.45% in DSR2.5, while the fraction of deformed regions decreases correspondingly. This can be attributed to the progressive development of shear bands and bending bands at higher speed ratios, which, within an appropriate range, increase the KAM value and the fraction of low-angle grain LAGBs. These deformation-induced defects provide the driving force for local recovery and continuous dynamic recrystallization, thereby promoting the gradual increase in recrystallized fraction at higher differential speed ratios [21]. However, a higher recrystallized fraction does not necessarily indicate better microstructural uniformity or deformation compatibility. Although the DSR2.5 sample exhibits the finest grain structure and the highest recrystallized fraction, its KAM value and dislocation density decrease slightly. This suggests that the microstructural evolution at this speed ratio is no longer governed solely by defect accumulation but gradually shifts toward structural reconstruction. Therefore, an intermediate differential speed ratio is more favorable for maintaining a balance between defect storage and microstructural stability.

**Figure 6:**
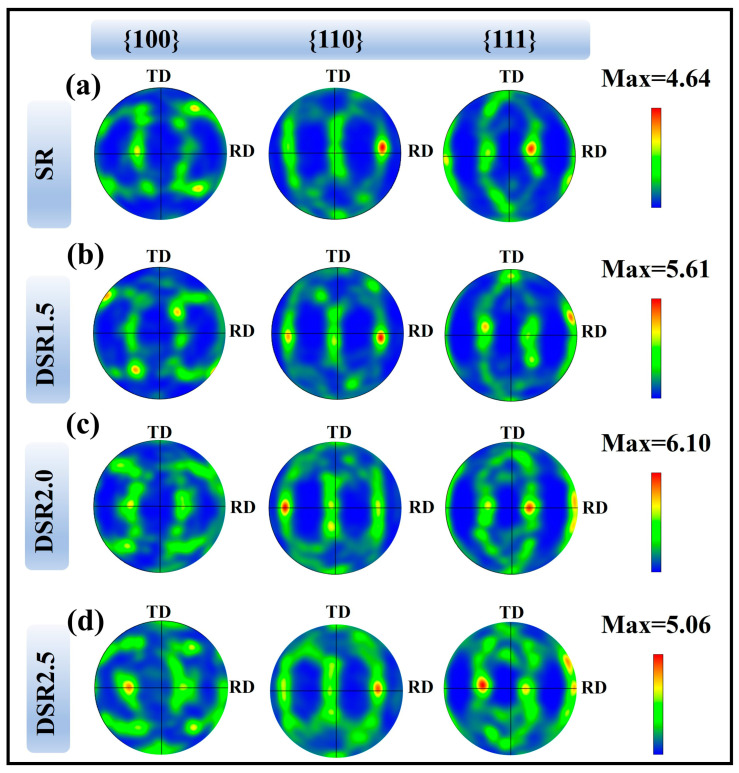
Pole figures of 7075 aluminum alloy processed at differential speed ratios: (**a**) SR; (**b**) DSR1.5; (**c**) DSR2.0; and (**d**) DSR2.5.

Figure 6 shows the {100}, {110}, and {111} pole figures of the 7075 aluminum alloy processed at different differential speed ratios. After rolling and aging, the {110} and {111} pole figures exhibit relatively continuous and symmetric orientation bands, indicating the development of a typical FCC rolling texture mainly associated with Brass, Copper, and S components [22]. In contrast, the {100} pole figure shows relatively low orientation intensity, suggesting that Cube texture is not significantly strengthened and that recrystallization remains limited after rolling. With an increasing differential speed ratio, the pole density first increases and then decreases. The SR sample shows a relatively dispersed orientation distribution, whereas the DSR1.5 and DSR2.0 samples exhibit increasingly concentrated high-density regions, indicating that differential speed rolling promotes grain rotation and orientation concentration within an appropriate speed-ratio range. Among all conditions, DSR2.0 shows the strongest orientation concentration, especially in the {111} pole figure near the RD, suggesting that an intermediate speed ratio is most favorable for shear-induced texture strengthening. When the speed ratio is further increased to 2.5, the high-density regions become broader and more diffuse, reflecting intensified local shear heterogeneity and partial orientation scattering at the highest speed ratio [23].

**Figure 7:**
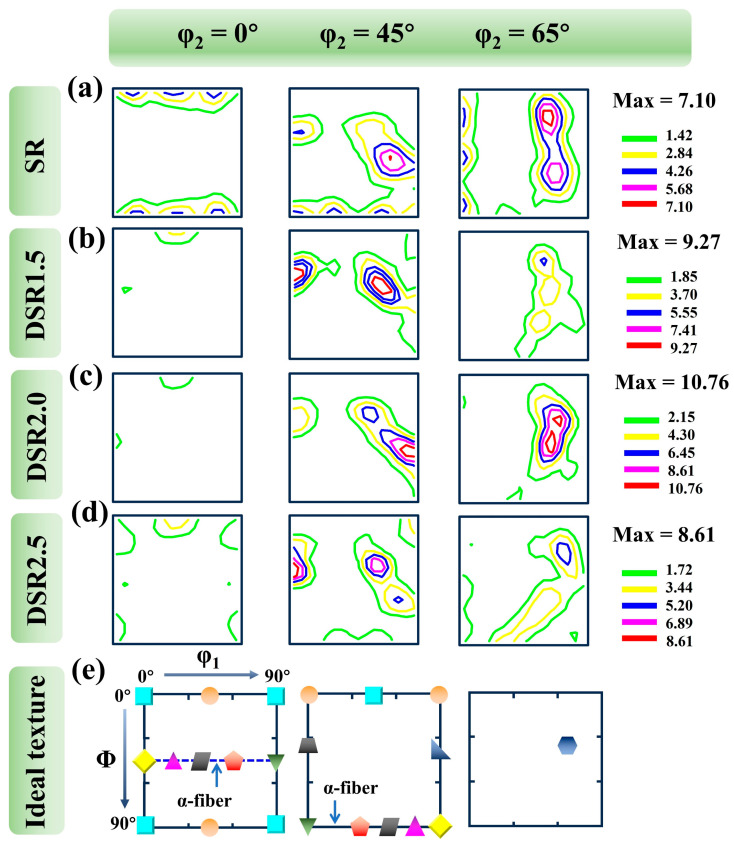
ODF figures of 7075 aluminum alloy processed at differential speed ratios: (**a**) SR; (**b**) DSR1.5; (**c**) DSR2.0; (**d**) DSR2.5; and (**e**) ideal texture.

Figure 7 presents the ODF sections of the 7075 aluminum alloy processed at differential speed ratios. Table 2 lists the common ideal texture components in face-centered cubic (FCC) metals [24]. All rolled samples exhibit typical FCC deformation textures mainly distributed along the α-fiber [25]. In the SR sample, the overall texture intensity is relatively low, with a maximum value of 7.10. The φ_2_ = 45° section shows intensity concentration mainly near the Copper {112} ⟨111⟩ and M {113} ⟨110⟩ components, whereas the φ_2_ = 65° section exhibits a pronounced peak near the S {123} ⟨634⟩ component, indicating the formation of a typical FCC rolling texture under plane-strain compression.

When the differential speed ratio increases to 1.5, the maximum texture intensity rises to 9.27. The peak near Copper {112} ⟨111⟩ becomes more distinct and elongated, and the α-fiber-related orientations are also strengthened. Meanwhile, the S component in the φ_2_ = 65° section remains clearly visible and becomes more extended. These changes indicate that the additional shear strain introduced by DSR1.5 begins to accelerate lattice rotation and promotes the development of shear-related deformation texture on the basis of the conventional rolling texture [10].

At DSR2.0, the overall texture intensity further increases and reaches a maximum value of 10.76. The Copper {112} ⟨111⟩ and S {123} ⟨634⟩ components are further intensified, and the peak distribution becomes more concentrated, especially in the φ_2_ = 45° and φ_2_ = 65° sections. This indicates that the coupling between compressive deformation and redundant shear deformation is strongest under the DSR2.0 condition. Such an enhanced and relatively stable deformation texture is beneficial not only for maintaining a high level of orientation-related strengthening but also for improving deformation compatibility and delaying plastic instability, thereby enabling the synergistic optimization of strength and ductility.

When the differential speed ratio is further increased to 2.5, the overall texture intensity decreases to 8.61. Although the FCC deformation texture is still retained, the peak distribution becomes broader and more diffuse. Weak orientation features appear near Cube {001} ⟨100⟩ and Rt-Cube {001} ⟨110⟩, suggesting that local recovery or incipient recrystallization may occur at the highest speed ratio. Meanwhile, the weakening and spreading of the Copper- and S-related peaks indicate that excessive shear can no longer effectively strengthen the deformation texture; instead, it aggravates local deformation heterogeneity. The resulting texture dispersion and strength reduction are unfavorable for sustaining work hardening and homogeneous plastic deformation.

### 3.3. Influence of Differential Speed Ratio on the Intragranular Precipitation Behavior of 7075 Aluminum Alloy

**Figure 8:**
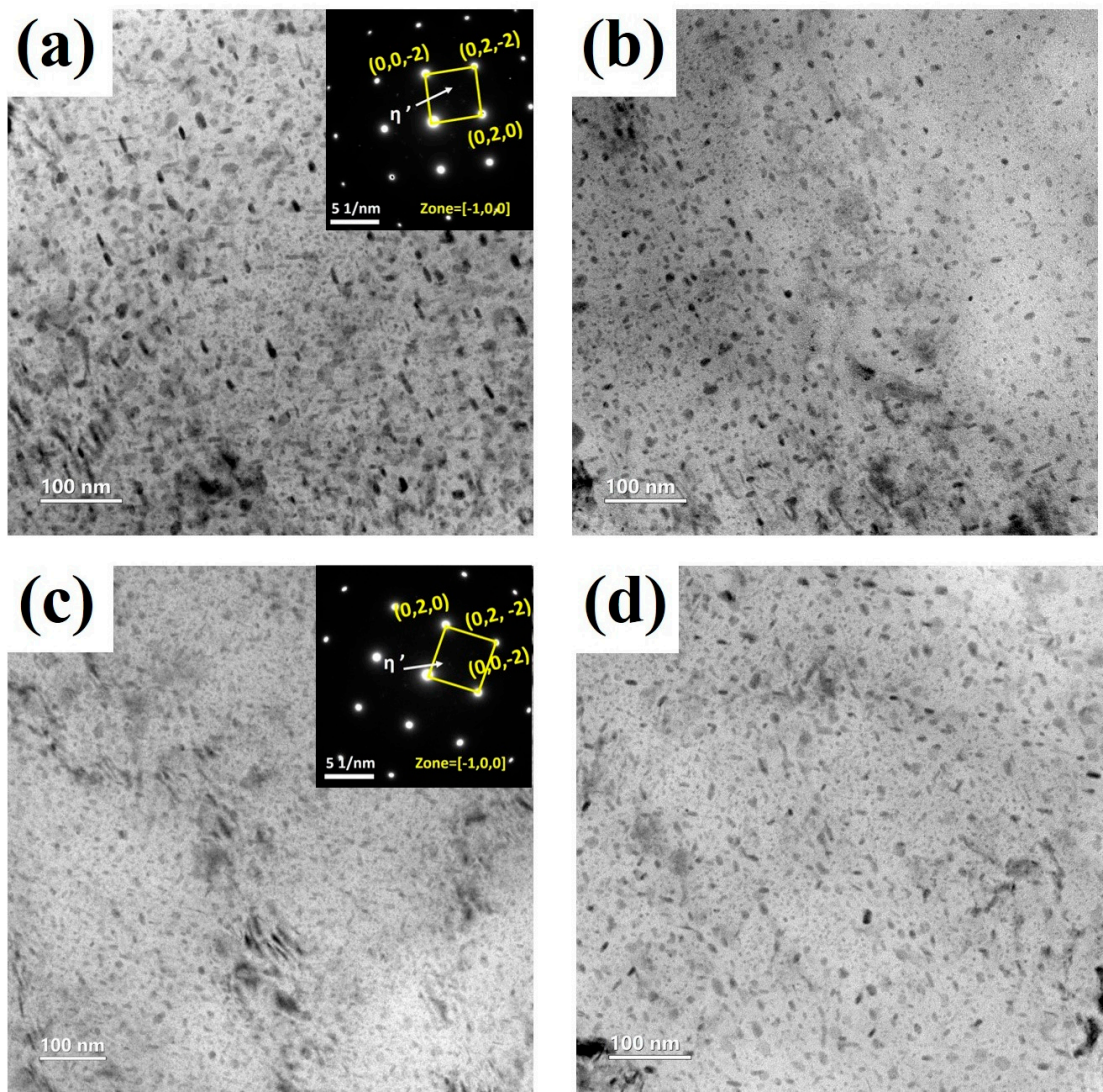
TEM morphologies of intragranular precipitates in 7075 aluminum alloy processed at differential speed ratios: (**a**) SR; (**b**) DSR1.5; (**c**) DSR2.0; and (**d**) DSR2.5.

Figure 8 shows the TEM morphologies of intragranular precipitates in the 7075 aluminum alloy processed at differential speed ratios, and the corresponding precipitate statistics are summarized in Table 3. As a typical precipitation-strengthened Al-Zn-Mg-Cu alloy, 7075 aluminum alloy generally follows the sequence of supersaturated solid solution → GP zones → η′ → η during aging [25,26]. As shown in Figure 8, a large number of fine precipitates are distributed within the grains in all samples, mainly exhibiting dot-like and short rod-like morphologies [27]. According to the SAED patterns shown in the insets, distinct diffraction spots can be observed at the 1/3 and 2/3 positions of {−220}, which are characteristic of the semi-coherent η′ phase [28]. This indicates that after rolling followed by aging at 120 °C for 24 h, the intragranular precipitates are still dominated by the η′ strengthening phase.

**Figure 9:**
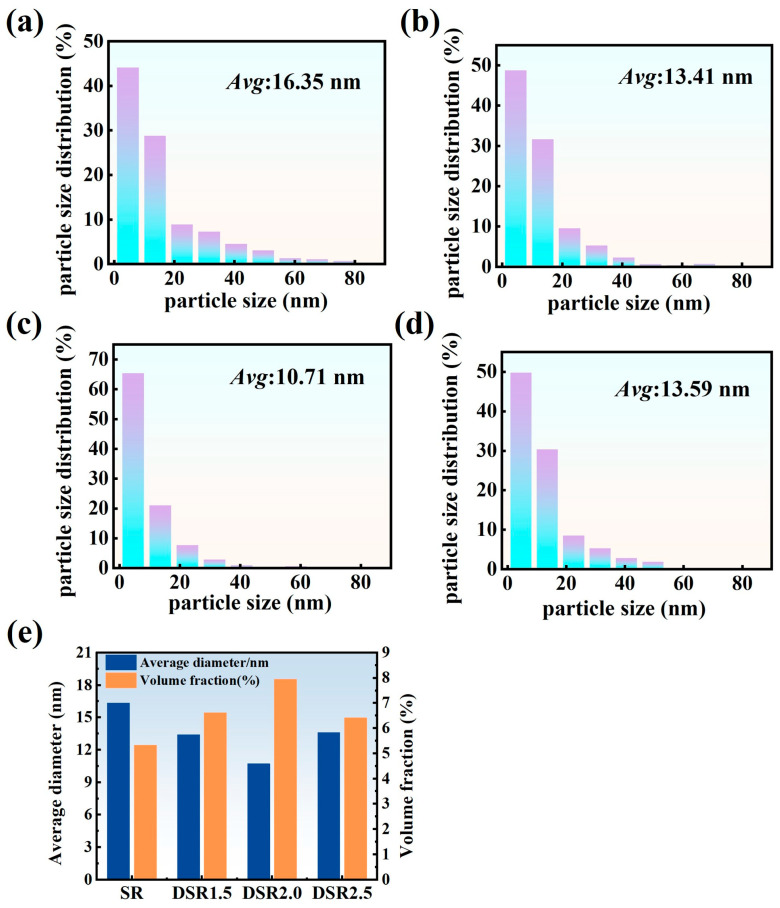
Intragranular precipitate size distributions and corresponding statistical comparison of 7075 aluminum alloy processed at differential speed ratios: (**a**) SR; (**b**) DSR1.5; (**c**) DSR2.0; (**d**) DSR2.5; and (**e**) comparison of average precipitate diameter and volume fraction.

Figure 9 shows the size distributions and corresponding statistical comparison of intragranular precipitates in the 7075 aluminum alloy processed at different differential speed ratios. (The precipitate size and number density were statistically analyzed using Image-Pro Plus 6.0 software, Media Cybernetics, Silver Spring, MD, USA). In the SR sample, the precipitates are relatively coarse and heterogeneously distributed, with an average size of 16.35 nm. Correspondingly, the precipitate number density is the lowest, at 1.27 × 10^23^ m^−3^, and the volume fraction is only 5.33%, suggesting that the precipitation state in the SR sample is characterized by relatively coarse but sparsely distributed intragranular precipitates.

As the differential speed ratio increases to 1.5 and 2.0, the precipitates become markedly finer and more uniformly distributed, and the average size decreases to 13.41 nm and 10.71 nm, respectively, with the most pronounced refinement being observed in the DSR2.0 sample. Meanwhile, the precipitate number density increases to 4.52 × 10^23^ m^−3^ in DSR2.0, while the volume fraction rises to 7.95%. The corresponding size distributions also shift toward the finer-size range, indicating that the additional shear deformation introduced by an intermediate differential speed ratio promotes the formation of finer and denser η′ precipitates. This can be attributed to the increased defect density and stored energy, which provide faster diffusion paths and more heterogeneous nucleation sites for solute atoms [29].

When the differential speed ratio is further increased to 2.5, the average precipitate size increases to 13.59 nm, while the number density and volume fraction decrease accordingly, indicating a certain coarsening tendency at the highest speed ratio. Notably, the rolling temperature under the DSR2.5 condition also reaches the highest value of 88.6 ± 5.9 °C, suggesting more pronounced local heat accumulation during processing. The stronger frictional heating and local temperature rise at the higher speed ratio may accelerate atomic diffusion and promote dislocation rearrangement, dynamic recovery, and partial release of stored energy, thereby weakening the high-energy defect structures that originally favor the high-density nucleation of fine η′ precipitates [30]. As a result, some of the already formed fine precipitates undergo competitive growth, whereas the formation of new effective nuclei is suppressed.

### 3.4. Mechanical Properties

**Figure 10:**
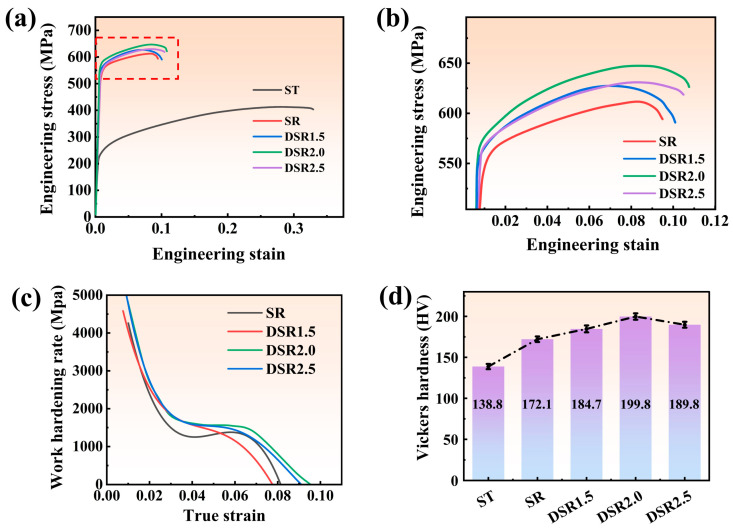
Effect of different speed ratios on the mechanical properties of 7075 aluminum alloy: (**a**) engineering stress–strain curves; (**b**) enlarged view of (**a**); (**c**) work-hardening curves; and (**d**) hardness.

Figure 10 and Table 4 indicate that the differential speed ratio significantly affects the mechanical properties of 7075 aluminum alloy. Compared with the ST condition, all rolled and aged samples exhibit markedly increased strength and hardness, accompanied by a pronounced decrease in elongation. The SR sample shows a UTS, YS, and EL of 611.23 MPa, 554.56 MPa, and 9.48%, respectively. As the differential speed ratio increases to 1.5 and 2.0, both strength and ductility are simultaneously improved, and the DSR2.0 sample exhibits the best comprehensive mechanical performance, with the UTS, YS, and EL reaching 648.43 MPa, 582.26 MPa, and 10.75%, respectively. When the speed ratio is further increased to 2.5, both strength and elongation show a slight decline, indicating a marginal effect on property improvement at an excessively high-speed ratio.

The strain-hardening exponent (*n*) was determined by fitting the true stress–true strain data using the Hollomon equation [31]:

(2) $$\sigma =K\varepsilon^{n}$$

where σ is the true stress, ε is the true strain, and K is the strength coefficient. To ensure fitting reliability, only the uniform plastic deformation stage was considered, and the fitting was performed over the true strain range of 0.01–0.09. The *n* value was obtained from the slope of the linear fit in the lnσ-lnε plot [32]. The hardness and work-hardening behavior are consistent with the tensile results. The Vickers hardness increases from 138.8 HV in the ST state to 172.1 HV in the SR sample, reaches a maximum of 199.8 HV in the DSR2.0 sample, and then decreases slightly to 189.8 HV in DSR2.5. Compared with the SR sample, the DSR samples maintain relatively higher work-hardening rates during the intermediate and late deformation stages. In particular, the DSR2.0 sample exhibits the highest strain-hardening exponent, with an *n* value of 0.101, indicating a stronger capability to sustain uniform deformation and delay plastic instability.

It is noteworthy that, under the DSR2.0 condition, the increase in strength is not accompanied by a loss of ductility. This can be attributed to the most favorable combination of refined banded grains and the highest fraction of LAGBs in DSR2.0. These LAGBs can act as effective buffering interfaces, coordinating continuous dislocation motion and alleviating severe local dislocation pile-up that would otherwise promote premature crack initiation [33]. In addition, the intragranular precipitates in DSR2.0 are extremely fine. Fine precipitates may locally improve the coordination of dislocation motion, thereby promoting more homogeneous slip and enhanced ductility [34]. This improved microstructural homogeneity is ultimately reflected in the macroscopic tensile response, thereby enabling a synergistic improvement in strength and ductility.

### 3.5. Strengthening Mechanisms

As a typical heat-treatable alloy, the strengthening behavior of 7075 aluminum alloy can be interpreted using a synergistic multi-mechanism model. Assuming that the individual strengthening contributions act independently, the yield strength can be expressed as follows [35]:

(3) $$\sigma_{\mathrm{tot}}=\sigma_{\mathrm{SS}}+\sigma_{\mathrm{Or}}+\sigma_{\mathrm{HP}}+\sigma_{\rho }$$

where σ*_SS_*, σ*_Or_*, σ*_HP_*, and σ*_ρ_* represent the contributions from solid-solution strengthening, precipitation strengthening, grain-boundary strengthening, and dislocation strengthening, respectively. Since all samples in the present study were solution-treated at 470 °C for 1.5 h prior to aging, the contribution of solid-solution strengthening is neglected here. The grain-boundary strengthening contribution, σ*_HP_*, can be estimated using the Hall–Petch relationship [36]:

(4) $$\sigma_{HP}=k/\sqrt{d}$$

where *k* is the Hall–Petch constant, reported to be 0.12 MPa·m^1/2^ for 7075 aluminum alloy, and *d* is the average grain size. The dislocation strengthening contribution can be expressed as follows [37]:

(5) $$\sigma_{\rho }=MCGb\sqrt{\rho }$$

where *M* is the Taylor factor (3.06 for FCC), *C* is a constant (0.3), *G* is the shear modulus (26 GPa), *b* is the Burgers vector (2.86 × 10^−10^ m), and *ρ* is the dislocation density.

Precipitation strengthening in 7xxx aluminum alloys is generally governed by either the Orowan bypassing mechanism or the dislocation shearing mechanism [38]. In the present study, the average precipitate size is much larger than the reported critical radius of 2.1 nm [39]. Therefore, the strengthening contribution of the intragranular precipitates is assumed to be dominated by the Orowan bypassing mechanism. The corresponding yield-strength increment can be written as follows:

(6) $$\sigma_{or}=M\frac{0.4Gb}{\pi \sqrt{1-v}}\cdot \frac{\ln(2/b)}{\lambda_{p}}$$

where *b* is the Burgers vector; *ν* is Poisson’s ratio (0.33); *r* is the average precipitate radius; and *λ_p_* is the interparticle spacing, which can be estimated as follows [38]:

(7) $$\lambda_{p}=4r\;\left(\sqrt{\frac{\pi }{4f_{v}}}-1\right)$$

where $f_{v}$ is the precipitate volume fraction. In this study, $f_{v}$ was estimated from TEM statistics according to the following [40]:

(8) $$f_{v}=\frac{N\pi d_{t}^{2}t}{2As(\phi +dt)}$$

where *N* is the number of precipitates counted in the analyzed TEM area, *d_t_* is the average precipitate diameter, *t* is the characteristic precipitate thickness, *As* is the analyzed area, and ϕ is the TEM foil thickness.

**Figure 11:**
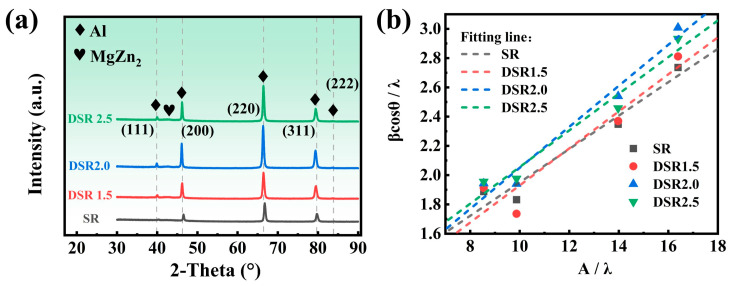
(**a**) XRD patterns under differential speed ratios; (**b**) Williamson–Hall plot.

To overcome the spatial limitations of EBSD, XRD was employed in this study to analyze the full width at half maximum (FWHM) of diffraction peaks, thereby enabling a more reliable evaluation of the dislocation density within subgrains, which is critical for assessing the contribution of dislocation strengthening [20]. The XRD patterns of samples processed at differential speed ratios (Figure 11a) exhibit characteristic peaks corresponding to the (111), (200), (220), and (311) planes, and the corresponding FWHM values and Bragg angles are summarized in Table 5.

The dislocation density (*ρ*) was calculated using the Williamson–Hall (W-H) method [41]:

(9) $$\rho =\frac{3\varepsilon \sqrt{2\pi }}{bd}$$

The microstrain (*ε*) was obtained from the XRD data according to the W-H equation [42]:

(10) $$\beta \frac{\cos\theta }{\lambda }=\frac{K}{d}+4\varepsilon \frac{\sin\theta }{\lambda }$$

where β is the FWHM of the diffraction peak (in radians), θ is the Bragg angle, λ is the wavelength of the Cu Kα radiation (0.154 nm), and *K* is the shape factor (assumed as 0.9). The microstrain (*ε*) is determined from the slope of the linear fit of expression (10) plotted against 4 sinθ/λ. Using Origin 2021 Pro software for linear fitting, the ε values for SR to 2.5 are 0.114, 0.127, 0.140, and 0.125, respectively, as shown in Figure 11b. The microstrain and dislocation density of different samples are summarized in Table 6. Both parameters reach their maximum values under the DSR2.0 condition, indicating that an intermediate differential speed ratio is most favorable for dislocation multiplication and stored-energy accumulation induced by redundant shear deformation. In contrast, both values decrease slightly in the DSR2.5 sample, suggesting the occurrence of local recovery at the highest speed ratio. In addition, the XRD results are in good agreement with the evolution of the KAM distribution obtained from EBSD: from SR to DSR2.0, the overall KAM level increases together with the fraction of low-angle grain boundaries, whereas both the KAM value and dislocation density decrease slightly under the DSR2.5 condition. The consistent trends obtained from these two methods mutually support each other, thereby enhancing the reliability of the dislocation-evolution analysis.

The calculated strengthening contributions are summarized in Table 7. Precipitation strengthening is the dominant contribution in all samples, ranging from 284.27 to 334.49 MPa, while dislocation strengthening is the second largest contributor. In contrast, the contribution of grain-boundary strengthening is relatively limited. From SR to DSR2.0, all three strengthening terms increase simultaneously. The increase in grain-boundary strengthening is associated with the progressive grain refinement during DSR, whereas the enhancement in dislocation strengthening reflects the higher dislocation density and stored energy introduced by shear deformation. More importantly, the marked increase in precipitation strengthening indicates that an appropriate differential speed ratio promotes the formation of finer and denser intragranular precipitates, thereby maximizing the precipitation strengthening effect. Therefore, the superior strength of the DSR2.0 sample can be mainly attributed to the synergistic effects of microstructural refinement, enhanced defect storage, and optimized precipitate distribution. When the differential speed ratio is further increased to 2.5, σ*_HP_* continues to increase slightly, whereas both σ*_ρ_* and σ*_or_* decrease. This indicates that an excessively high-speed ratio can no longer effectively enhance defect accumulation. Instead, stronger local shear deformation and the higher deformation-induced temperature rise promote partial dislocation rearrangement and recovery, thereby reducing the density of high-energy defect sites. Meanwhile, a certain coarsening tendency of the precipitates further weakens the precipitation strengthening effect. It should be noted that the sum of the calculated strengthening terms does not exactly match the measured yield strength. This discrepancy is reasonable because the present model mainly considers the dominant strengthening contributions and does not fully include the intrinsic matrix strength, residual solid-solution strengthening, texture-related anisotropy, or the interactions among different strengthening mechanisms [43]. Therefore, the present calculation should be regarded as a semi-quantitative comparison among different DSR conditions.

### 3.6. Tensile Fracture Morphology and Fracture Mechanism Analysis

**Figure 12:**
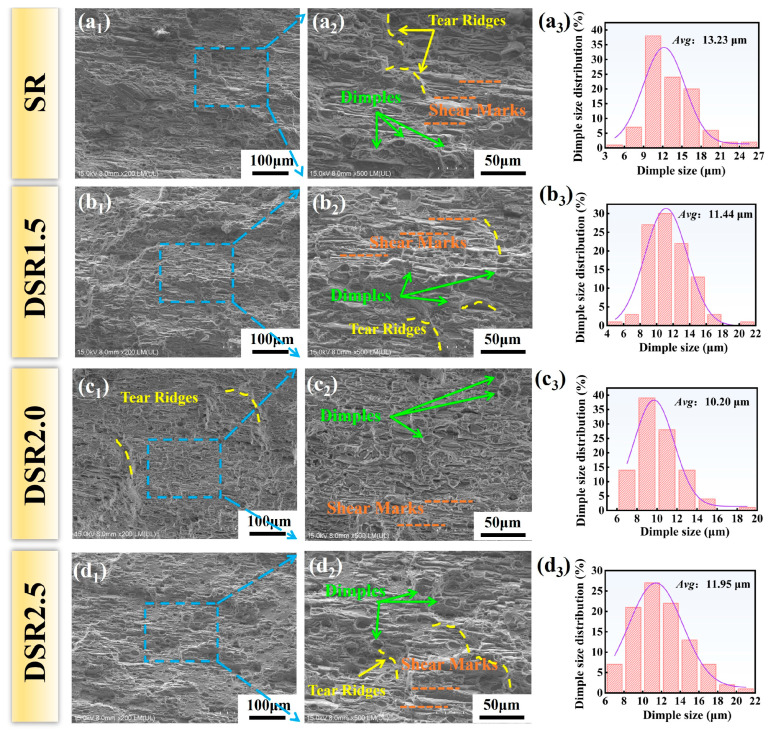
Tensile fracture morphologies and dimple size distributions of 7075 aluminum alloy processed at differential speed ratios: (**a_1_**–**d_1_**) low-magnification fracture morphologies; (**a_2_**–**d_2_**) enlarged local fracture features; and (**a_3_**–**d_3_**) dimple size distributions.

Figure 12 shows the tensile fracture morphologies and dimple size distributions of 7075 aluminum alloy processed at different differential speed ratios. All samples exhibit a mixed ductile fracture mode characterized by dimples, tear ridges, and shear marks, indicating that fracture mainly proceeds through the nucleation, growth, and coalescence of microvoids, accompanied by local shear tearing [44]. Quantitative analysis further reveals that the average dimple size first decreases and then increases with increasing differential speed ratio, reaching a minimum value of 10.20 at DSR2.0. These results indicate that the differential speed ratio has a significant effect on fracture morphology and fracture stability.

The evolution of dimple morphology directly reflects the variation in macroscopic ductility and is essentially governed by the underlying microstructural evolution (Figure 3 and Figure 4). From SR to DSR2.0, the average grain size continuously decreases from 9.26 μm to 7.42 μm. Such a refined grain structure, together with a dense substructure, promotes the formation and more uniform distribution of microvoid nucleation sites, thereby leading to finer and deeper dimples [10]. The DSR2.0 sample exhibits the finest and most homogeneous dimple morphology, with the smallest average dimple diameter of 10.20 μm. Although tear ridges and shear marks are still present, their degree of localization is relatively limited. This fracture feature is consistent with the higher elongation and stronger work-hardening capability of the sample, and also explains, from a micromechanical perspective, why DSR2.0 exhibits the best overall mechanical performance and the highest elongation.

By contrast, when the differential speed ratio is further increased to 2.5, more pronounced shear marks and tear ridges reappear, and some dimples become elongated or shallow, indicating intensified shear localization and local strain concentration. Although the grain size is further slightly reduced, the extremely high shear strain induces strong macroscopic shear bands, while the slight decrease in the KAM value and LAGB fraction suggests the occurrence of partial recovery and structural instability. These microstructural features provide preferential paths for rapid microvoid linkage during tensile deformation, thereby weakening fracture stability and leading to the simultaneous deterioration of ductility and strength [45]. Overall, an intermediate differential speed ratio, particularly DSR2.0, is most favorable for obtaining a finer and more uniform dimple structure together with a more stable fracture process.

## 4. Conclusions

This study investigated the influence of differential speed ratio on the microstructural evolution, texture development, precipitation behavior, and mechanical properties of 7075 aluminum alloy. The main conclusions are as follows:

(1) Increasing the differential speed ratio enhances redundant shear deformation, leading to progressive grain refinement and more pronounced deformation substructures. In particular, the DSR2.0 sample exhibits the highest KAM value and the largest fraction of LAGBs, indicating the strongest defect accumulation and the most pronounced substructure refinement. By contrast, DSR2.5 already shows signs of partial recovery and local recrystallization.
(2) The differential speed ratio markedly affects texture evolution and precipitation behavior. The deformation texture is strengthened first and then weakened slightly at the highest speed ratio, while the intragranular η′ precipitates first become finer and then coarsen slightly. Among all conditions, DSR2.0 exhibits the strongest deformation texture and the most favorable precipitation state.
(3) Under the same rolling reduction and aging conditions, the mechanical properties first improve and then decline slightly with increasing differential speed ratio. The DSR2.0 sample achieves the best strength–ductility balance, with a high-yield strength, ultimate tensile strength, elongation, and hardness of 582.26 MPa, 648.43 MPa, 10.75%, and 199.8 HV, respectively.
(4) Strengthening analysis indicates that precipitation strengthening is dominant, while dislocation strengthening also makes an important contribution. The superior mechanical performance of DSR2.0 results from the synergistic effects of refined grain structure, high defect storage, stable deformation texture, and fine, uniformly distributed η′ precipitates. In contrast, excessive shear at DSR2.5 reduces microstructural stability and slightly deteriorates the overall mechanical performance.

## Figures and Tables

**Table 1:** Chemical composition of the 7075 alloy (wt.%).

Element	Zn	Mg	Cu	Fe	Cr	Mn	Al
Measured	6.01	2.54	1.84	0.12	0.22	0.20	Bal.
Reference	5.1~6.1	2.1~2.9	1.2~2.0	<0.5	0.18~0.28	<0.3	Bal.

**Table 2:** Common texture components in FCC and their corresponding Miller indices and Euler angles.

Texture Component	Symbol	Miller Indices	Euler Angle (°)		
			φ_1_	Φ	φ_2_
Cube	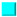	{001} <100>	0	0	0
Rt-Cube	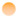	{001} <110>	45	0	0
Brass	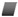	{110} <112>	35	45	0
Goss	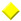	{110} <100>	0	45	0
Rt-Goss	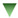	{110} <110>	90	45	0
Copper	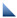	{112} <111>	90	35	45
F	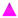	{110} <115>	74	90	0
S	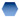	{123} <634>	59	37	63
M	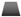	{113} <110>	0	25	45
P	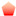	{110} <455>	65	45	0

**Table 3:** Summary of intragranular precipitate statistics for 7075 aluminum alloy processed at differential speed ratios.

Sample	SR	DSR1.5	DSR2.0	DSR2.5
number of measured precipitates	497	514	523	488
Average diameter (nm)	16.35	13.41	10.71	13.59
Number density (m^−3^)	1.27 × 10^23^	2.34 × 10^23^	4.52 × 10^23^	2.21 × 10^23^
Volume fraction (%)	5.33	6.62	7.95	6.41

**Table 4:** Mechanical properties of 7075 aluminum alloy processed at different speed ratios.

Sample	UTS/MPa	YS/MPa	EL/%	Hardness/HV	*n*
ST	422.54 ± 3.1	244.92 ± 3.5	31.25 ± 0.3	138.8 ± 3.3	—
SR	611.23 ± 3.8	554.56 ± 3.6	9.48 ± 0.4	172.1 ± 3.5	0.088
DSR1.5	627.55 ± 4.6	565.92 ± 4.7	10.10 ± 0.5	184.7 ± 4.4	0.091
DSR2.0	648.43 ± 4.3	582.26 ± 4.1	10.75 ± 0.4	199.8 ± 3.6	0.101
DSR2.5	632.48 ± 4.5	572.23 ± 3.6	10.44 ± 0.5	189.8 ± 4.9	0.097

**Table 5:** FWHM values of various crystal planes at differential speed ratios.

Sample	FWHM			
	(111)	(200)	(220)	(311)
SR	0.308	0.305	0.429	0.543
DSR1.5	0.312	0.289	0.433	0.558
DSR2.0	0.317	0.323	0.464	0.597
DSR2.5	0.319	0.329	0.449	0.582

**Table 6:** Microstrain and dislocation density at differential speed ratios.

Sample	SR	DSR1.5	DSR2.0	DSR2.5
d (μm)	9.26	8.035	7.421	7.093
ε	0.114	0.127	0.140	0.125
*Ρ* (m^−2^)	3.24 × 10^14^	4.16 × 10^14^	4.97 × 10^14^	4.64 × 10^14^

**Table 7:** Calculated strengthening contributions of 7075 aluminum alloy processed at differential speed ratios.

Samples	*σ_HP_*	*σ_ρ_*	*σ_or_*
SR	39.43	122.87	284.27
DSR1.5	42.33	139.23	307.73
DSR2.0	44.05	152.18	334.49
DSR2.5	45.06	147.04	301.09

## Data Availability

The original contributions presented in the study are included in the article, further inquiries can be directed to the corresponding author.

## Abbreviations

The following abbreviations are used in this manuscript:

SR	symmetric rolling
DSR	differential speed rolling
YS	yield strength
UTS	ultimate tensile strength
EL	elongation
ICP-OES	inductively coupled plasma optical emission spectrometry
SPD	severe plastic deformation
ECAP	equal-channel angular pressing
HPT	high-pressure torsion
RD	rolling direction
FE-SEM	field-emission scanning electron microscope
EDS	energy-dispersive X-ray spectroscopy
ND	normal direction
PF	pole figures
IPF	inverse pole figures
KAM	kernel average misorientation
ODF	orientation distribution function
XRD	X-ray diffraction
ST	solution treatment
HAGBs	high-angle grain boundaries
LAGBs	low-angle grain boundaries
SBs	shear bands
KBs	kink bands
GND	geometrically necessary dislocation density
FWHM	full width at half maximum
